# Molecular evidence for natural hybridization in the mangrove fern genus *Acrostichum*

**DOI:** 10.1186/1471-2229-13-74

**Published:** 2013-05-01

**Authors:** Rongshu Zhang, Ting Liu, Wei Wu, Yunqin Li, Lifang Chao, Lishi Huang, Yelin Huang, Suhua Shi, Renchao Zhou

**Affiliations:** 1 State Key Laboratory of Biocontrol and Guangdong Key Laboratory of Plant Resources, Sun Yat-sen University, Guangzhou, 510275, China; 2 Key Laboratory of Plant Resources Conservation and Sustainable Utilization, South China Botanical Garden, Chinese Academy of Sciences, Guangzhou, 510650, China; 3 Experimental Center of Fundamental Teaching, Zhuhai Campus, Sun Yat-sen University, Zhuhai, 519802, China

**Keywords:** Acrostichum, Chloroplast DNA, Ferns, Mangroves, Natural hybridization, Nuclear genes

## Abstract

**Background:**

Natural hybridization is prevalent in ferns, and plays an important role in fern evolution and speciation. In the Indo West-Pacific region, the mangrove fern genus *Acrostichum* consists of two largely sympatric species, *A. aureum* and *A. speciosum*. Although there has been no report of interspecific hybridization before, we found some individuals morphologically intermediate between them in Guangdong and Hainan, China, for the first time, which were suspected to be hybrids. In this study, we aimed to test the hypothesis of natural hybridization between *A. aureum* and *A. speciosum* in Guangdong and Hainan using three low-copy nuclear genes. A chloroplast intergenic spacer was used to infer the hybridization direction once the hybrid status was confirmed. In addition, we examined spore shapes and germination for these taxa.

**Results:**

Both *A. aureum* and *A. speciosum* showed a low level of polymorphism at all three nuclear genes; however, they were well separated at these loci. At both locations, each individual of the putative hybrid showed additivity in chromatograms at all sites where the two species showed fixed differences. Haplotype analysis at all three nuclear genes indicated that each individual of the putative hybrid possessed two haplotypes, matching with those of *A. aureum* and *A. speciosum*, respectively. Sequencing of the chloroplast *trn*V-*trn*M regions showed that *A. aureum* differed from *A. speciosum* by eleven nucleotide substitutions and three indels (insertions/deletions), and all sampled individuals of the putative hybrid had the identical sequences with *A. speciosum.* Compared with *A. aureum* and *A. speciosum*, the putative hybrid had much reduced spore germination rate.

**Conclusions:**

Sequence data of the three nuclear genes provide compelling evidence for natural hybridization between *A. aureum* and *A. speciosum*, and all the hybrid individuals are likely F1s. The hybridization is unidirectional and *A. speciosum* is the maternal parent of the hybrid based on the assumption of maternal inheritance of chloroplast DNA. Human disturbance on mangrove habitats may facilitate the establishment of hybrids of *Acrostichum*.

## Background

Natural hybridization is a common phenomenon in plants and has a significant role in plant evolution [1,2]. Depending on the interaction of intrinsic and extrinsic factors, interspecific hybridization can lead to formation of stable genetic lineages in the forms of polyploid or homoploid hybrid species (reviewed in [3]), reinforcement of premating isolation as a response to maladaptive hybrids [4], and gene transfer through genetic recombination or introgression [5-8]. Hybridization occurs more prevalent in ferns than flowering plants and a wide spectrum of fern species are found to be derived from interspecific hybridization [2,9-13]. In the past several decades, many cases of natural hybridization in ferns have been identified (e.g. [10,14-18]). In this study, we will focus on natural hybridization between two species in the mangrove fern genus *Acrostichum*.

Consisting of about 70 species from about 20 families [19], mangroves are a special group of plants that inhabit the intertidal zones of tropical and subtropical coasts. *Acrostichum* L., the only fern genus of mangroves, comprises three species, namely, *A. aureum* L., *A. speciosum* Willd. and *A. danaeifolium* Langsd. & Fish [20]. Among the three species, *A. speciosum* is restricted in the Indo West-Pacific (IWP) region and *A. danaeifolium* is confined to the Atlantic East-Pacific (AEP) region, while *A. aureum* is widely distributed in both regions [20,21]. Species of *Acrostichum* are usually called mangrove ferns.

**Figure 1:**
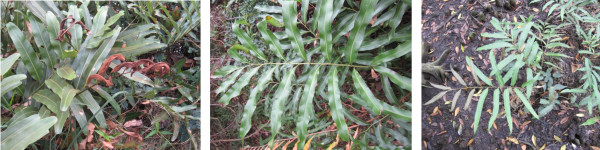
**Morphological illustrations for*A. aureum*(left), the putative hybrid (middle), and*A. speciosum*(right).**

In China, *A. aureum* is a common species, ranging from Hainan through Guangdong and Guangxi to Fujian and Taiwan. In contrast, *A. speciosum* is a rare species, occurring in only two locations, Wenchang, Hainan and Zhanjiang, Guangdong (W. Wang, unpublished data). Both *A. aureum* and *A. speciosum* occur on the landward edge of mangrove swamps, however, they show different ecological preferences to light and salinity. *A. aureum* is often encountered as a river or creek plant where fresh water influence is strong [20,22,23]. It also behaves like a light demanding fern, and often can be found in deteriorated or disturbed mangrove areas [24,25]. In contrast, *A. speciosum* is usually found at the mangrove understory, and just at the margins of high intertidal zones. These areas are frequently inundated by tides and usually are shady [20,26]. In fields, the two species are easily distinguished based on frond shape and texture. The frond of *A. speciosum* narrows gradually to a pointed tip, while that of *A. aureum* has a broadly rounded end (Figure 1). Additionally, the frond of *A. aureum* is thickly coriaceous, while that of *A. speciosum* is papery. It seems that the textures of the two species conform to their ecological preferences to light.

During our field trips in Wenchang, Hainan and Zhanjiang, Guangdong, where the two species coexist, we found some individuals morphologically intermediate between *A. aureum* and *A. speciosum*. These individuals have leathery fronds similar to *A. aureum*, and gradually narrowed and pointed tips like *A. speciosum*. Most, if not all, individuals were found very close to the shrimp ponds, where the natural mangrove habitats have been seriously disturbed or destroyed. In China, we haven’t found any individuals of this kind in other areas where only *A. aureum* occurs. With intermediate morphology and overlapping geographic distribution, these individuals are suspected to be interspecific hybrids between *A. aureum* and *A. speciosum*.

Because plants often show a high degree of morphological plasticity in response to various environmental factors, and the intermediate morphology can arise from forces other than hybridization, morphological criteria alone for identifying natural hybrids are inadequate. Conventional approaches such as morphological analysis, chromosome number and pairing behavior, and allozyme assay have been widely used to study hybridization in ferns (e.g. [11,15,27,28], these methods, however, either fail to provide convincing conclusions or are time and labor consuming, especially when the sample size is large. Recently, single or low-copy nuclear genes have proven successful in identifying hybrids in plants [14,29-35]. In this study, we aimed to test the hypothesis of natural hybridization between *A. aureum* and *A. speciosum* using the sequences of three low-copy nuclear genes. Once the hybrid status was confirmed, a chloroplast DNA fragment was used to determine the maternal parent of these hybrid individuals, because chloroplast DNA is usually maternally transmitted in ferns [36]. In addition, we examined the spore shapes and germination rates for the three taxa to see if the putative hybrid has reduced fertility relative to its putative parents.

## Results

At the three nuclear genes, almost all samples of *A. aureum* and *A. speciosum*, and most samples of the putative hybrid, could be directly sequenced and clear sequences were obtained. For each gene, both *A. aureum* and *A. speciosum* exhibited limited sequence variation (see below for details). However, these two species were highly diverged at all three nuclear genes and one chloroplast DNA fragment.

### Sequences analysis of *cam* genes in *A. aureum*, *A. speciosum* and the putative hybrid

**Figure 2:**
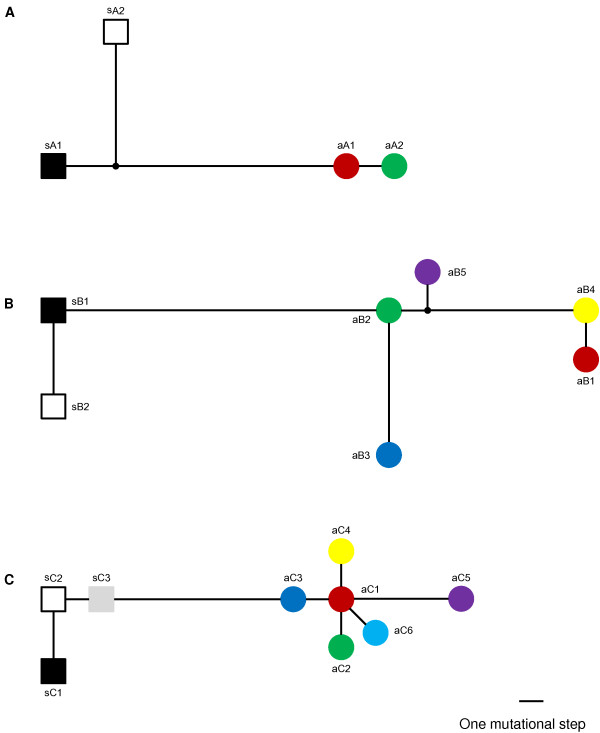
**Haplotype networks of*cam*(A),*gapCp1*(B) and*gapCp2*(C) genes for*A. speciosum*and*A. aureum*.** Color circles represent haplotypes of *A. aureum*, and black, white and grey squares represent haplotypes of *A. speciosum*. a and s in the haplotype names mean *A. aureum* and *A. speciosum*, respectively. Small black circles represent hypothetical or unsampled haplotypes. Mutational steps are shown by the length of the connecting lines.

**Figure 3:**
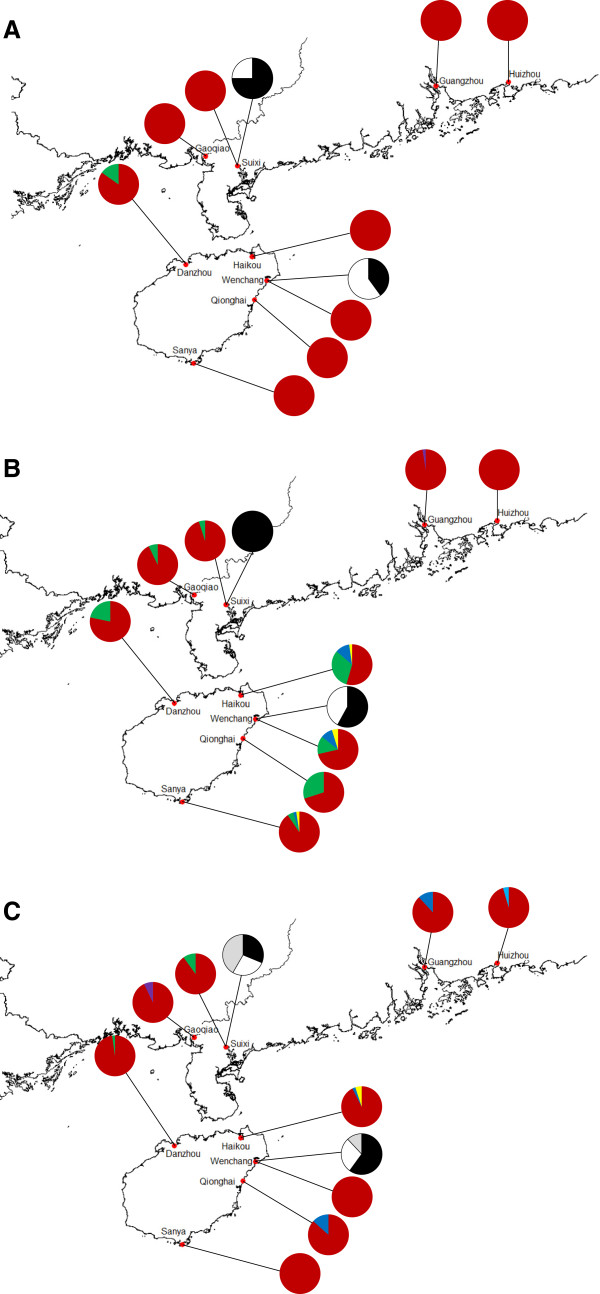
**Haplotype distribution of the three nuclear genes*cam*(A),*gapCp1*(B) and*gapCp2*(C) in each population of *A. aureum*and*A. speciosum*.** Color codes correspond to those in Figure 2.

The partial sequences of the *cam* gene we analyzed were 453 bp in length in the three taxa of *Acrostichum*. There were nine fixed nucleotide substitutions between *A. aureum* and *A. speciosum*. For the putative hybrid, all individuals showed chromatogram additivity at these nine fixed sites between *A. aureum* and *A. speciosum*. Both species showed a low level of haplotype diversity at the *cam* gene, with two haplotypes being observed in each species (Figure 2A). All but the Danzhou population of *A. aureum* were monomorphic and shared the common haplotype aA1, and the other haplotype aA2 differed from haplotype aA1 by one mutation (Figure 3A). Both populations of *A. speciosum* possessed haplotypes sA1 and sA2, although the frequencies of the two haplotypes were different between them (Figure 3A). For the putative hybrid, seven individuals from Wenchang and nine individuals from Suixi showed a combination of haplotype aA1 of *A. aureum* and haplotype sA1 of *A. speciosum* (Table 1; Additional file 1: Table S1). Haplotypes of the remaining six individuals were a combination of haplotype aA1 of *A. aureum* and haplotype sA2 of *A. speciosum* (Table 1).

**Table 1:** **Haplotype combination for the putative hybrid of*Acrostichum*at three nuclear genes**

**Gene**	Haplotype combination of the putative hybrid*	**Number of individuals of the putative hybrid**	
		Wenchang	Suixi
*cam*	aA1/sA1	7	9
	aA1/sA2	3	3
*gapCp1*	aB1/sB1	6	8
	aB1/sB2	2	0
	aB2/sB1	1	0
	aB2/sB2	1	0
	UN/sB1	0	4
*gapCp2*	aC1/sC1	9	6
	aC1/sC2	1	5
	UN/sC2	0	1

*UN: Unique haplotype to the putative hybrid

Designations of haplotypes were identical with those in Figure 2.

**Table 2:** **Sampling details of*A. aureum*,*A. speciosum*and their putative hybrid**

**Taxon**	**Sampling location**	**Number of individuals**	**Voucher**
*A. aureum*	Qinglan Bay,Wenchang, Hainan, China	21	Zhou 120601
	Yalong Bay, Sanya, Hainan, China	21	Zhou 110801
	Tanmen, Qionghai, Hainan, China	15	Zhou 110802
	Dongzhai Bay, Haikou, Hainan, China	21	Zhou 110803
	Guangcun, Danzhou, Hainan, China	23	Zhou 110804
	Renshan, Huizhou,Guangdong, China	22	Ouyang 110901
	Nansha, Guangzhou, Guangdong, China	21	Zhou 111001
	Suixi, Zhanjiang, Guangdong, China	13	Zhou 110901
*A. speciosum*	Gaoqiao, Zhanjiang,Guangdong, China	21	Zhou 110904
	Qinglan Bay, Wenchang, Hainan, China	25	Zhou 120602
	Suixi, Zhanjiang, Guangdong, China	24	Zhou 110902
Putative hybrid	Qinglan Bay, Wenchang, Hainan, China	10	Zhou 120603
	Suixi, Zhanjiang, Guangdong, China	12	Zhou 110903

### Sequences analysis of *gapCp1* genes in *A. aureum*, *A. speciosum* and the putative hybrid

The partial *gapCp1* genes were 601 bp in the three taxa after sequence alignment. *A. aureum* and *A. speciosum* differed in eleven fixed nucleotide substitutions and one fixed 5-bp indel (insertion/deletion). All individuals of the putative hybrid showed chromatogram additivity at these eleven fixed sites and one indel region. Six haplotypes were identified among all samples of *A. aureum*, and haplotype aB1 was dominant across all populations (Figure 2B). Only Huizhou population was monomorphic, and the number of haplotypes varied from two to four in other populations (Figure 3B). In contrast, *A. speciosum* exhibited a much lower level of haplotype diversity, with only two haplotypes being detected (Figure 2B). Wenchang population had two haplotypes (sB1 and sB2), while Suixi population had only one haplotype sB1 (Figure 3B). For the putative hybrid from Wenchang, six individuals showed a combination of haplotype aB1 of *A. aureum* and sB1 of *A. speciosum*, two individuals showed a combination of haplotype aB1 of *A. aureum* and haplotype sB2 of *A. speciosum*, one individual showed a combination of haplotype aB2 of *A .aureum* and haplotype sB1of *A. speciosum*, and one individual showed a combination of haplotype aB2 of *A. aureum* and haplotype sB2 of *A. speciosum* (Table 1; Additional file 1: Table S1). For the putative hybrid from Suixi, eight individuals showed a mix of haplotype aB1 of *A. aureum* and haplotype sB1 of *A. speciosum*, and haplotypes of the remaining four individuals were a combination of haplotype sB1 of *A. speciosum* and a haplotype unique to the putative hybrid, which differed by one mutation from the common haplotype aB1 of *A. aureum* (Table 1).

### Sequence analysis of *gapCp2* genes in *A. aureum*, *A. speciosum* and the putative hybrid

The aligned length of *gapCp2* genes in the three taxa was 490 bp. There were seven fixed nucleotide substitutions between *A. aureum* and *A. speciosum*. For the putative hybrid, all individuals showed chromatogram additivity at these seven fixed sites. In the haplotype analysis, six and three haplotypes were detected for *A. aureum* and *A. speciosum*, respectively (Figure 2C). The number of haplotypes varied from one to three in the populations of *A. aureum* and haplotype aC1 was dominant across all populations (Figure 2C, Figure 3C). In *A. speciosum*, three haplotypes (sC1, sC2 and sC3) were shared by the two populations (Figure 3C). For the putative hybrid from both locations, haplotypes of all but one individual were a combination of haplotype aC1 of *A. aureum* and haplotype sC1 or sC2 of *A. speciosum* (Table 1; Additional file 1: Table S1). Haplotypes of the special individual were a combination of haplotype sC2 and a unique haplotype to the putative hybrid, which differed by one mutation from the common hyplotype aC1 of *A. aureum* (Table 1).

### Sequence analysis of chloroplast *trn*V*-trn*M in *A. aureum*, *A. speciosum* and the putative hybrid

The chloroplast *trn*V-*trn*M sequences of *A. aureum* and *A. speciosum* were both 975 bp in length. No sequence variation was detected within each species. There were eleven nucleotide substitutions and three indels in this region between *A. aureum* and *A. speciosum*. All sampled individuals of the putative hybrid from Wenchang and Suixi had the same *trn*V-*trn*M sequences as *A. speciosum*.

### Spore morphology and germination of *A. aureum*, *A. speciosum* and the putative hybrid

The spores of *A. aureum*, *A. speciosum* and the putative hybrid were all globose and similar in size (diameter: 40–55 μm). However, the putative hybrid showed a much lower spore germination rate (20.3%) compared with *A. aureum* (79.8%) and *A. speciosum* (65.4%).

## Discussion

### Genetic variation in *A. aureum* and *A. speciosum*

Both *A. aureum* and *A. speciosum* are widespread in the Indo West-Pacific region; in China, however, *A. aureum* is much more common than *A. speciosum*. Our results indicate that both species harbor a low level of polymorphism at the three nuclear genes and a chloroplast intergenic spacer. Many populations have only one or two haplotypes at all of the loci examined, suggesting severe diversity loss or recent colonization in these species. It is well recognized that repeated population bottlenecks due to fluctuation of sea levels in the Pleistocene glaciation caused diversity loss in mangrove species [37]. Low levels of polymorphism in the two mangrove ferns are consistent with this historical demography. However, *A. aureum* and *A. speciosum* are well separated based on network analysis of the three nuclear genes and sequence divergence at the chloroplast fragment, indicating that the two species have diverged for a long time.

### Molecular identification of natural hybridization between *A. aureum* and *A. speciosum*

Although *A. aureum* and *A. speciosum* overlap largely in geographic distribution in the Indo West-Pacific region, there has been no report of natural hybridization between them before. In this study, we used three low-copy nuclear genes to test the hypothesis of natural hybridization between them. Multiple individuals of *A. aureum* and *A. speciosum*, as well as their putative hybrid, have been used for this purpose. Although one haplotype unique to the putative hybrid is observed at each of the *gapCp1* and *gapCp2* genes, it is very close to the common haplotype of *A. aureum* (one mutational step). Unsampled polymorphisms in *A. aureum* may cause this pattern. This finding is not surprising given comparatively high diversity at these two genes in *A. aureum*. All individuals of the putative hybrid show perfect chromatogram additivity at each of the three nuclear genes, providing convincing evidence for natural hybridization between *A. aureum* and *A. speciosum*. The much reduced spore germination rate in the putative hybrid provides another line of evidence for this hypothesis. Moreover, these hybrid individuals are all likely F1s, because all of them show biparental inheritance at all three randomly selected nuclear genes. At the *gapCp1* gene, haplotype sB2 of *A. speciosum* is unique to Wenchang population, and hybrids with this haplotype are found in only that location, suggesting the hybridization should arise locally. Given chloroplast DNA is predominantly maternally inherited in ferns [36], the chloroplast data indicate that *A. speciosum* might be the maternal parent of these hybrid individuals and the hybridization was unidirectional. Unidirectional hybridization in *Acrostichum* may owe to differential abundance of parental species. At both sites we studied, *A. aureum* is much more abundant than *A. speciosum*. *A. aureum* may thus produce much more mobile sperms, which have more chances to fertilize the egg of *A. speciosum*. In addition, strong prezygotic and (or) postzygotic barriers in the other direction (e.g. incompatibility of foreign gamete) and selection pressures from the environments on hybrid progeny may be other possibilities [6,16,18,38,39].

### Factors contributing to natural hybridization between *A. aureum* and *A. speciosum*

In ferns, meiosis results in spores which give rise to gametophytes, and just in this situation sexual reproduction can occur [40]. The tiny and short-lived gametophytic generation plays an important role in the reproduction and dispersal ecology of ferns [41]. There is considerable overlap in geographic distribution between *A. aureum* and *A. speciosum*, especially in Southeast Asia and North Australia [19,20]. Although these two species have distinctive habitat preferences, the spores of both species can be dispersed by sea currents [20] and their gametophytes may come into contact as a consequence of spore dispersal. Zygotes may thus establish via the fusion of gametes respectively originating from gametophytes of these two species, providing spatial chances for hybridization.

### The consequences of hybridization between *A. aureum* and *A. speciosum*

Despite frequent occurrence of hybridization in *Acrostichum*, it seems that all hybrid individuals we sampled are simple F1s. The restriction of hybrids to F1s appears to be a general phenomenon in ferns [9,42], and interspecific hybrids are sterile mostly because chromosomes do not behave normally in the process of spore production, resulting in abortive spores [14,42]. In this study, although the hybrid is very similar to its parental species in spore shape and size, its spore germination rate is much reduced. Post-germination selection may further cause the absence of later-generation hybrids. The persistence of only sterile F1 hybrids is guaranteed by recurrent hybridization, which is to some extent in accord with that the hybrid is only found in places where its parental species overlap.

It is well known that hybridization followed by polyploidy is important in fern evolution and speciation [10,43-45]. Sterile F1 hybrids can obtain the reproductive capacity through genome doubling [10,45]. The chromosome number of these two *Acrostichum* species are both 2n=60 [46,47]. So far, we have no data on chromosome number of these hybrid individuals, so further survey on this is deserved.

It is widely believed that endogenous and exogenous factors both play important roles in selection against hybrids [2,5,48]. We have not found any hybrids of *Acrostichum* in typical habitats of both parental species, implying selection against hybrids in the habitats of parental species. The hybrids, however, are frequently observed in the intermediate environments, for example, newly deforested sites for fish ponds, suggesting higher fitness of these hybrids in this kind of habitat. Hence, human disturbance on mangrove habitats may facilitate the establishment of hybrids of *Acrostichum*.

Growing at the interface between land and sea, mangroves are of great ecological significance [49,50]. They protect and stabilize coastlines, yield commercial forest products, furnish nursery and spawning areas for commercially important coastal fishes and so on [49,51]. *Acrostichum*, the only fern genus of mangroves, is one of the important mangrove components in both the IWP and AEP regions. *A. aureum* is a pioneer species of mangroves and it grows rapidly after mangrove forests have been destroyed and forms extensive dense thickets of about 3 to 4 m height, particularly in the more elevated inland areas less frequently being inundated by tides [52]. *A. speciosum*, an understory plant, shows special adaptation to shady habitats. Their hybrid is usually found on the areas disturbed by human such as aquacultural ponds. All of them could affect the establishment and growth of other mangrove species, especially in the areas destroyed by human and natural disasters [52,53], so further study about the ecological significance of the hybrid is important for mangrove management and protection. In particular, it is valuable to conduct well-designed studies on fitness of the hybrid in a variety of habitats, including parental, intermediate, as well as novel habitats.

## Conclusions

*Acrostichum aureum* and *A. speciosum* are the only two mangrove fern species in the Indo West-Pacific region and they show largely overlapping distribution although their habitats are distinct. In this study, we identified natural hybridization between them for the first time, based on sequence data of three nuclear genes. Interestingly, all the hybrid individuals are likely F1s, suggesting no gene flow between the two species. Therefore, the two species can maintain genetic integrity despite hybridization. Chloroplast DNA shows that the hybridization is unidirectional, with *A. speciosum* as the maternal parent, and this might be caused by much higher abundance of *A. aureum* at the study sites. The two species usually occur in differentiated habitats, which might be an effective barrier for interspecific hybridization. Human disturbance on mangrove habitats, however, may bring their spores into contact and thus facilitate the establishment of hybrids of *Acrostichum*. The impact of hybridization in this genus on mangrove ecosystems should be investigated in future.

## Methods

### Plant sampling

Plants of *A. aureum* and *A. speciosum* and their putative hybrid were sampled from two locations in China: Qinglan Bay, Wenchang, Hainan, and Suixi, Zhanjiang, Guangdong. Frond tissues of *A. aureum* and *A. speciosum* and the putative hybrid were collected in plastic bags with silica gels for DNA extraction. In addition, we also collected samples of *A. aureum* from Sanya, Qionghai, Danzhou and Haikou of Hainan and Huizhou, Guangzhou, and Zhanjiang of Guangdong, where only *A. aureum* occurs and no putative hybrid individuals were found. Voucher specimens were deposited in the Herbarium of Sun Yat-sen University (SYS). Table 2 listed the details of the samples. We also collected mature fertile fronds of the three taxa in Qinglan Bay, Wenchang, Hainan, to examine the morphology and germination of their spores.

### Spore morphology and germination

We collected mature sporangia (partially opened) under a dissecting microscope and transferred them to a drop of water on the microscope slide, tapped gently on the top of the cover glass to scatter the spores. The shape of the spores was examined under a Leica DMI4000 B microscope. Because we found that mature spores released from the sporangia of the three taxa could directly germinate on the fertile fonds after the fertile fonds were placed in petri dishes containing distilled water for three days, we compared the spore germination rates for the three taxa by this means. For each taxon, more than 200 mature spores from multiple fertile fonds were used to calculate the germination rate. Spores were considered be germinated with the emergence of the rhizoid.

### Loci studied

Three low-copy nuclear genes, namely, *cam*, *gapCp1* and *gapCp2*, and one chloroplast intergenic spacer, *trn*V*-trn*M, were used in this study. We first used the universal *cam* primers for angiosperms [54] to test the feasibility in *Acrostichum*, and when we got the sequences from an individual of *A. aureum*, we designed specific *cam* primers *camb*-F (5′ AAGTTCTTTGGTTGTGATGCAA 3′) and *camb*-R (5′ GATGGTGAGTGTGTGCATTTG 3′) for *Acrostichum*. With regard to the *gapCp* gene, we first used universal *gapCp* primers in ferns [55] to test their feasibility in *Acrostichum*, and two bright bands with different length were amplified. We sequenced these two bands and found both of them were members of the *gapCp* gene family based on sequence homology. We then designated them as *gapCp1* and *gapCp2* respectively and designed two pairs of specific primers, *gapCp1*-F (5′ GCTGCATATACATAGGGACATCG 3′) and *gapCp1*-R (5′ CAAAGTGATCATTCAAAGCAATG 3′), *gapCp2*-F (5′ CGAGAAGAATCTTTAGCCAACCT 3′) and *gapCp2*-R (5′ GTCGTACCAAGCCACAAGTTTC 3′), to amplify them. The chloroplast *trn*V*-trn*M was amplified using the universal primers *trn*V and *trn*M [56].

### DNA extraction, PCR and sequencing

Total DNA was extracted from dried frond tissues using the CTAB method according to Doyle and Doyle (1987) [57]. PCR was conducted using KOD-FX DNA polymerase (Toyobo, Osaka, Japan) with the following conditions: initial denaturation at 94°C for 4 min, followed by 30 cycles of 98°C for 10 s, corresponding annealing temperature (54°C for *gapCp2* and *trn*V*-trn*M, 58°C for *gapCp1*, and 62°C for *cam*) for 30 s, and 68°C for 1 min. PCR products were purified using the Pearl Gel Extraction Kit (Pearl Biotech, Guangzhou, China). Purified PCR products were directly sequenced on an ABI 3730 DNA Analyzer with the BigDye Terminator Cycle Sequencing Ready Reaction Kit (Applied Biosystems, Foster City, California, USA). Intra-individual length polymorphism for the nuclear genes could cause failure of direct sequencing from the polymorphic sites. In addition, some individuals, mainly from the putative hybrid, had superimposed chromatograms at multiple sites of the nuclear genes, and the haplotypes could not be inferred. Under these circumstances, cloning sequencing was used to phase the haplotypes. Ligations were conducted using the pMD18-T&A cloning kit (Takara, Dalian, China). Eight positive colonies for each individual were selected for sequencing. The sequences were deposited in GenBank with accession numbers JX575141-JX575175 (Additional file 1: Table S2).

### Sequence analyses

All sequences of the three nuclear genes and one chloroplast intergenic spacer were aligned and compared in SeqMan™ (DNASTAR, Madison, Wisconsin, USA). Haplotype inference of these three nuclear genes was implemented with PHASE v2.1 [58,59]. Haplotype network was constructed for each gene using Network 4.6.1.0 [60] with the median-joining algorithm [61].

## Competing interests

The authors declare no competing interests.

## Authors’ contributions

The experimental design was conceived by RCZ and RSZ and all experiments were performed by RSZ and TL. Data was analyzed by RSZ and RCZ with assistance from WW, YL, LC, LH, YH, and SS. This paper was written by RCZ, RSZ, WW and SS. All authors read and approved the final manuscript.

## Supplementary Material

Additional file 1: Table S1 Genotype information at the three nuclear genes for all sampled individuals of the three taxa in *Acrostichum.***Table S2** Haplotypes at the three nuclear genes and their GenBank accession numbers for the three taxa in *Acrostichum.* Click here for file
