# Supplementary material for: Molecular evidence for natural hybridization in the mangrove fern genus Acrostichum
Source: BMC Plant Biol. 2013 May 1;13:74. doi: 10.1186/1471-2229-13-74 (PMC3668252; doi:10.1186/1471-2229-13-74)
Supplement: Additional file 1: Table S1 — Genotype information at the three nuclear genes for all sampled individuals of the three taxa in Acrostichum.Table S2 Haplotypes at the three nuclear genes and their GenBank accession numbers for the three taxa in Acrostichum. [file 1471-2229-13-74-S1.doc]

**Supplementary Tables**

***Supplementary Table 1*** *Genotype information at the three nuclear genes for all sampled individuals of the three taxa in Acrostichum*

| Species | Population | Individual No. | *cam* | *gapcp1* | *gapcp2* |
| --- | --- | --- | --- | --- | --- |
| *A. aureum* | Wenchang | 1 | aA1/aA1 | aB1/aB3 | aC1/aC1 |
|  |  | 2 | aA1/aA1 | aB1/aB1 | aC1/aC1 |
|  |  | 3 | aA1/aA1 | aB1/aB1 | aC1/aC1 |
|  |  | 4 | aA1/aA1 | aB1/aB1 | aC1/aC1 |
|  |  | 5 | aA1/aA1 | aB1/aB1 | aC1/aC1 |
|  |  | 6 | aA1/aA1 | aB1/aB1 | aC1/aC1 |
|  |  | 7 | aA1/aA1 | aB3/aB3 | aC1/aC1 |
|  |  | 8 | aA1/aA1 | aB1/aB2 | aC1/aC1 |
|  |  | 9 | aA1/aA1 | aB1/aB2 | aC1/aC1 |
|  |  | 10 | aA1/aA1 | aB1/aB3 | aC1/aC1 |
|  |  | 11 | aA1/aA1 | aB1/aB1 | aC1/aC1 |
|  |  | 12 | aA1/aA1 | aB1/aB2 | aC1/aC1 |
|  |  | 13 | aA1/aA1 | aB1/aB1 | aC1/aC1 |
|  |  | 14 | aA1/aA1 | aB1/aB4 | aC1/aC1 |
|  |  | 15 | aA1/aA1 | aB1/aB1 | aC1/aC1 |
|  |  | 16 | aA1/aA1 | aB1/aB1 | aC1/aC1 |
|  |  | 17 | aA1/aA1 | aB1/aB1 | aC1/aC1 |
|  |  | 18 | aA1/aA1 | aB1/aB4 | aC1/aC1 |
|  |  | 19 | aA1/aA1 | aB1/aB2 | aC1/aC1 |
|  |  | 20 | aA1/aA1 | aB1/aB1 | aC1/aC1 |
|  |  | 21 | aA1/aA1 | aB2/aB2 | aC1/aC1 |
|  | Sanya | 1 | aA1/aA1 | aB1/aB1 | aC1/aC1 |
|  |  | 2 | aA1/aA1 | aB1/aB1 | aC1/aC1 |
|  |  | 3 | aA1/aA1 | aB1/aB1 | aC1/aC1 |
|  |  | 4 | aA1/aA1 | aB1/aB1 | aC1/aC1 |
|  |  | 5 | aA1/aA1 | aB1/aB1 | aC1/aC1 |
|  |  | 6 | aA1/aA1 | aB1/aB1 | aC1/aC1 |
|  |  | 7 | aA1/aA1 | aB1/aB1 | aC1/aC1 |
|  |  | 8 | aA1/aA1 | aB1/aB1 | aC1/aC1 |
|  |  | 9 | aA1/aA1 | aB1/aB1 | aC1/aC1 |
|  |  | 10 | aA1/aA1 | aB1/aB1 | aC1/aC1 |
|  |  | 11 | aA1/aA1 | aB1/aB1 | aC1/aC1 |
|  |  | 12 | aA1/aA1 | aB1/aB2 | aC1/aC1 |
|  |  | 13 | aA1/aA1 | aB1/aB1 | aC1/aC1 |
|  |  | 14 | aA1/aA1 | aB1/aB1 | aC1/aC1 |
|  |  | 15 | aA1/aA1 | aB1/aB4 | aC1/aC1 |
|  |  | 16 | aA1/aA1 | aB1/aB1 | aC1/aC1 |
|  |  | 17 | aA1/aA1 | aB1/aB2 | aC1/aC1 |
|  |  | 18 | aA1/aA1 | aB1/aB1 | aC1/aC1 |
|  |  | 19 | aA1/aA1 | aB1/aB1 | aC1/aC1 |
|  |  | 20 | aA1/aA1 | aB1/aB1 | aC1/aC1 |
|  |  | 21 | aA1/aA1 | aB1/aB3 | aC1/aC1 |
|  | Qionghai | 1 | aA1/aA1 | aB1/aB2 | aC1/aC1 |
|  |  | 2 | aA1/aA1 | aB1/aB2 | aC1/aC3 |
|  |  | 3 | aA1/aA1 | aB1/aB1 | aC1/aC1 |
|  |  | 4 | aA1/aA1 | aB2/aB2 | aC1/aC1 |
|  |  | 5 | aA1/aA1 | aB1/aB1 | aC1/aC1 |
|  |  | 6 | aA1/aA1 | aB1/aB1 | aC1/aC3 |
|  |  | 7 | aA1/aA1 | aB1/aB1 | aC1/aC1 |
|  |  | 8 | aA1/aA1 | aB1/aB2 | aC1/aC1 |
|  |  | 9 | aA1/aA1 | aB1/aB1 | aC1/aC1 |
|  |  | 10 | aA1/aA1 | aB1/aB2 | aC1/aC1 |
|  |  | 11 | aA1/aA1 | aB1/aB1 | aC1/aC3 |
|  |  | 12 | aA1/aA1 | aB1/aB1 | aC1/aC1 |
|  |  | 13 | aA1/aA1 | aB1/aB2 | aC1/aC1 |
|  |  | 14 | aA1/aA1 | aB1/aB2 | aC1/aC1 |
|  |  | 15 | aA1/aA1 | aB1/aB2 | aC1/aC3 |
|  | Haikou | 1 | aA1/aA1 | aB1/aB4 | aC1/aC1 |
|  |  | 2 | aA1/aA1 | aB1/aB2 | aC1/aC1 |
|  |  | 3 | aA1/aA1 | aB1/aB1 | aC1/aC1 |
|  |  | 4 | aA1/aA1 | aB2/aB2 | aC1/aC1 |
|  |  | 5 | aA1/aA1 | aB1/aB2 | aC1/aC1 |
|  |  | 6 | aA1/aA1 | aB1/aB1 | aC1/aC1 |
|  |  | 7 | aA1/aA1 | aB1/aB3 | aC1/aC1 |
|  |  | 8 | aA1/aA1 | aB1/aB2 | aC1/aC1 |
|  |  | 9 | aA1/aA1 | aB1/aB2 | aC1/aC4 |
|  |  | 10 | aA1/aA1 | aB1/aB2 | aC1/aC1 |
|  |  | 11 | aA1/aA1 | aB1/aB2 | aC1/aC1 |
|  |  | 12 | aA1/aA1 | aB2/aB3 | aC1/aC1 |
|  |  | 13 | aA1/aA1 | aB1/aB1 | aC1/aC1 |
|  |  | 14 | aA1/aA1 | aB1/aB1 | aC1/aC3 |
|  |  | 15 | aA1/aA1 | aB2/aB3 | aC1/aC1 |
|  |  | 16 | aA1/aA1 | aB1/aB1 | aC1/aC4 |
|  |  | 17 | aA1/aA1 | aB1/aB3 | aC1/aC1 |
|  |  | 18 | aA1/aA1 | aB1/aB2 | aC1/aC1 |
|  |  | 19 | aA1/aA1 | aB1/aB2 | aC1/aC1 |
|  |  | 20 | aA1/aA1 | aB2/aB3 | aC1/aC1 |
|  |  | 21 | aA1/aA1 | aB1/aB2 | aC1/aC1 |
|  | Danzhou | 1 | aA1/aA1 | aB1/aB1 | aC1/aC1 |
|  |  | 2 | aA1/aA2 | aB1/aB1 | aC1/aC1 |
|  |  | 3 | aA1/aA1 | aB1/aB1 | aC1/aC1 |
|  |  | 4 | aA1/aA1 | aB1/aB1 | aC1/aC1 |
|  |  | 5 | aA1/aA1 | aB1/aB2 | aC1/aC1 |
|  |  | 6 | aA1/aA1 | aB1/aB1 | aC1/aC1 |
|  |  | 7 | aA1/aA1 | aB1/aB1 | aC1/aC1 |
|  |  | 8 | aA1/aA1 | aB1/aB1 | aC1/aC1 |
|  |  | 9 | aA1/aA2 | aB2/aB2 | aC1/aC1 |
|  |  | 10 | aA1/aA1 | aB1/aB2 | aC1/aC1 |
|  |  | 11 | aA1/aA1 | aB1/aB1 | aC1/aC1 |
|  |  | 12 | aA1/aA1 | aB1/aB1 | aC1/aC1 |
|  |  | 13 | aA1/aA1 | aB1/aB2 | aC1/aC1 |
|  |  | 14 | aA1/aA1 | aB2/aB2 | aC1/aC1 |
|  |  | 15 | aA1/aA1 | aB1/aB1 | aC1/aC1 |
|  |  | 16 | aA1/aA1 | aB1/aB2 | aC1/aC1 |
|  |  | 17 | aA2/aA2 | aB1/aB2 | aC1/aC1 |
|  |  | 18 | aA1/aA1 | aB1/aB1 | aC1/aC2 |
|  |  | 19 | aA1/aA1 | aB1/aB1 | aC1/aC1 |
|  |  | 20 | aA1/aA2 | aB1/aB2 | aC1/aC1 |
|  |  | 21 | aA1/aA1 | aB1/aB1 | aC1/aC1 |
|  |  | 22 | aA1/aA1 | aB1/aB1 | aC1/aC1 |
|  |  | 23 | aA2/aA2 | aB1/aB1 | aC1/aC1 |
|  | Huizhou | 1 | aA1/aA1 | aB1/aB1 | aC1/aC1 |
|  |  | 2 | aA1/aA1 | aB1/aB1 | aC1/aC1 |
|  |  | 3 | aA1/aA1 | aB1/aB1 | aC1/aC1 |
|  |  | 4 | aA1/aA1 | aB1/aB1 | aC1/aC1 |
|  |  | 5 | aA1/aA1 | aB1/aB1 | aC1/aC1 |
|  |  | 6 | aA1/aA1 | aB1/aB1 | aC1/aC1 |
|  |  | 7 | aA1/aA1 | aB1/aB1 | aC1/aC1 |
|  |  | 8 | aA1/aA1 | aB1/aB1 | aC1/aC1 |
|  |  | 9 | aA1/aA1 | aB1/aB1 | aC1/aC1 |
|  |  | 10 | aA1/aA1 | aB1/aB1 | aC1/aC1 |
|  |  | 11 | aA1/aA1 | aB1/aB1 | aC1/aC1 |
|  |  | 12 | aA1/aA1 | aB1/aB1 | aC1/aC1 |
|  |  | 13 | aA1/aA1 | aB1/aB1 | aC1/aC1 |
|  |  | 14 | aA1/aA1 | aB1/aB1 | aC1/aC1 |
|  |  | 15 | aA1/aA1 | aB1/aB1 | aC1/aC6 |
|  |  | 16 | aA1/aA1 | aB1/aB1 | aC1/aC6 |
|  |  | 17 | aA1/aA1 | aB1/aB1 | aC1/aC1 |
|  |  | 18 | aA1/aA1 | aB1/aB1 | aC1/aC1 |
|  |  | 19 | aA1/aA1 | aB1/aB1 | aC1/aC1 |
|  |  | 20 | aA1/aA1 | aB1/aB1 | aC1/aC1 |
|  |  | 21 | aA1/aA1 | aB1/aB1 | aC1/aC1 |
|  |  | 22 | aA1/aA1 | aB1/aB1 | aC1/aC1 |
|  | Guangzhou | 1 | aA1/aA1 | aB1/aB1 | aC1/aC1 |
|  |  | 2 | aA1/aA1 | aB1/aB1 | aC1/aC1 |
|  |  | 3 | aA1/aA1 | aB1/aB1 | aC1/aC1 |
|  |  | 4 | aA1/aA1 | aB1/aB1 | aC1/aC1 |
|  |  | 5 | aA1/aA1 | aB1/aB1 | aC1/aC1 |
|  |  | 6 | aA1/aA1 | aB1/aB1 | aC1/aC1 |
|  |  | 7 | aA1/aA1 | aB1/aB1 | aC1/aC1 |
|  |  | 8 | aA1/aA1 | aB1/aB1 | aC1/aC1 |
|  |  | 9 | aA1/aA1 | aB1/aB1 | aC1/aC1 |
|  |  | 10 | aA1/aA1 | aB1/aB1 | aC1/aC3 |
|  |  | 11 | aA1/aA1 | aB1/aB1 | aC1/aC1 |
|  |  | 12 | aA1/aA1 | aB1/aB1 | aC1/aC1 |
|  |  | 13 | aA1/aA1 | aB1/aB1 | aC1/aC3 |
|  |  | 14 | aA1/aA1 | aB1/aB1 | aC1/aC1 |
|  |  | 15 | aA1/aA1 | aB1/aB1 | aC1/aC1 |
|  |  | 16 | aA1/aA1 | aB1/aB1 | aC1/aC1 |
|  |  | 17 | aA1/aA1 | aB1/aB5 | aC1/aC3 |
|  |  | 18 | aA1/aA1 | aB1/aB1 | aC1/aC3 |
|  |  | 19 | aA1/aA1 | aB1/aB1 | aC1/aC3 |
|  |  | 20 | aA1/aA1 | aB1/aB1 | aC1/aC1 |
|  |  | 21 | aA1/aA1 | aB1/aB1 | aC1/aC1 |
|  | Suixi | 1 | aA1/aA1 | aB1/aB1 | aC1/aC1 |
|  |  | 2 | aA1/aA1 | aB1/aB1 | aC1/aC1 |
|  |  | 3 | aA1/aA1 | aB1/aB1 | aC1/aC1 |
|  |  | 4 | aA1/aA1 | aB1/aB2 | aC1/aC1 |
|  |  | 5 | aA1/aA1 | aB1/aB1 | aC1/aC2 |
|  |  | 6 | aA1/aA1 | aB1/aB1 | aC1/aC1 |
|  |  | 7 | aA1/aA1 | aB1/aB1 | aC1/aC1 |
|  |  | 8 | aA1/aA1 | aB1/aB1 | aC1/aC1 |
|  |  | 9 | aA1/aA1 | aB1/aB1 | aC1/aC1 |
|  |  | 10 | aA1/aA1 | aB1/aB1 | aC1/aC2 |
|  |  | 11 | aA1/aA1 | aB1/aB1 | aC1/aC1 |
|  |  | 12 | aA1/aA1 | aB1/aB1 | aC1/aC1 |
|  |  | 13 | aA1/aA1 | aB1/aB1 | aC1/aC1 |
|  | Gaoqiao | 1 | aA1/aA1 | aB1/aB1 | aC1/aC1 |
|  |  | 2 | aA1/aA1 | aB1/aB2 | aC1/aC1 |
|  |  | 3 | aA1/aA1 | aB1/aB1 | aC1/aC1 |
|  |  | 4 | aA1/aA1 | aB1/aB1 | aC1/aC1 |
|  |  | 5 | aA1/aA1 | aB1/aB1 | aC1/aC5 |
|  |  | 6 | aA1/aA1 | aB1/aB1 | aC1/aC1 |
|  |  | 7 | aA1/aA1 | aB1/aB1 | aC1/aC1 |
|  |  | 8 | aA1/aA1 | aB1/aB1 | aC1/aC1 |
|  |  | 9 | aA1/aA1 | aB1/aB1 | aC1/aC1 |
|  |  | 10 | aA1/aA1 | aB1/aB1 | aC1/aC1 |
|  |  | 11 | aA1/aA1 | aB1/aB1 | aC1/aC1 |
|  |  | 12 | aA1/aA1 | aB1/aB1 | aC1/aC1 |
|  |  | 13 | aA1/aA1 | aB1/aB1 | aC1/aC1 |
|  |  | 14 | aA1/aA1 | aB1/aB2 | aC1/aC1 |
|  |  | 15 | aA1/aA1 | aB1/aB1 | aC1/aC1 |
|  |  | 16 | aA1/aA1 | aB1/aB1 | aC1/aC1 |
|  |  | 17 | aA1/aA1 | aB1/aB1 | aC1/aC1 |
|  |  | 18 | aA1/aA1 | aB1/aB1 | aC1/aC1 |
|  |  | 19 | aA1/aA1 | aB1/aB1 | aC1/aC5 |
|  |  | 20 | aA1/aA1 | aB1/aB2 | aC1/aC1 |
|  |  | 21 | aA1/aA1 | aB1/aB1 | aC1/aC5 |
| 1. *speciosum* | Wenchang | 1 | sA2/sA2 | sB1/sB1 | sC1/sC1 |
|  |  | 2 | sA2/sA2 | sB1/sB1 | sC2/sC3 |
|  |  | 3 | sA1/sA2 | sB1/sB1 | sC1/sC2 |
|  |  | 4 | sA1/sA1 | sB1/sB1 | sC1/sC1 |
|  |  | 5 | sA1/sA1 | sB1/sB1 | sC1/sC1 |
|  |  | 6 | sA2/sA2 | sB1/sB1 | sC1/sC2 |
|  |  | 7 | sA1/sA1 | sB1/sB2 | sC1/sC2 |
|  |  | 8 | sA2/sA2 | sB1/sB1 | sC1/sC2 |
|  |  | 9 | sA2/sA2 | sB1/sB2 | sC1/sC1 |
|  |  | 10 | sA1/sA2 | sB1/sB2 | sC1/sC2 |
|  |  | 11 | sA1/sA1 | sB1/sB2 | sC1/sC2 |
|  |  | 12 | sA1/sA2 | sB1/sB2 | sC2/sC2 |
|  |  | 13 | sA1/sA1 | sB2/sB2 | sC1/sC2 |
|  |  | 14 | sA1/sA2 | sB1/sB1 | sC1/sC3 |
|  |  | 15 | sA1/sA2 | sB1/sB1 | sC1/sC3 |
|  |  | 16 | sA1/sA1 | sB2/sB2 | sC1/sC1 |
|  |  | 17 | sA1/sA2 | sB1/sB1 | sC2/sC3 |
|  |  | 18 | sA1/sA2 | sB1/sB1 | sC2/sC3 |
|  |  | 19 | sA1/sA1 | sB1/sB2 | sC1/sC1 |
|  |  | 20 | sA1/sA2 | sB1/sB1 | sC1/sC2 |
|  |  | 21 | sA1/sA1 | sB1/sB1 | sC1/sC1 |
|  |  | 22 | sA1/sA2 | sB1/sB1 | sC1/sc3 |
|  |  | 23 | sA1/sA1 | sB1/sB1 | sC1/sC1 |
|  |  | 24 | sA1/sA1 | sB1/sB2 | sC1/sc2 |
|  |  | 25 | sA1/sA2 | sB1/sB2 | sC1/sC1 |
|  | Suixi | 1 | sA1/sA1 | sB1/sB1 | sC3/sC3 |
|  |  | 2 | sA1/sA2 | sB1/sB1 | sC1/sC3 |
|  |  | 3 | sA1/sA2 | sB1/sB1 | sC1/sC3 |
|  |  | 4 | sA1/sA2 | sB1/sB1 | sC1/sC3 |
|  |  | 5 | sA1/sA1 | sB1/sB1 | sC2/sC2 |
|  |  | 6 | sA1/sA1 | sB1/sB1 | sC2/sC3 |
|  |  | 7 | sA1/sA2 | sB1/sB1 | sC1/sC3 |
|  |  | 8 | sA1/sA1 | sB1/sB1 | sC2/sC2 |
|  |  | 9 | sA1/sA1 | sB1/sB1 | sC2/sC2 |
|  |  | 10 | sA1/sA1 | sB1/sB1 | sC1/sC3 |
|  |  | 11 | sA1/sA1 | sB1/sB1 | sC2/sC3 |
|  |  | 12 | sA1/sA1 | sB1/sB1 | sC3/sC3 |
|  |  | 13 | sA1/sA1 | sB1/sB1 | sC2/sC2 |
|  |  | 14 | sA1/sA1 | sB1/sB1 | sC2/sC3 |
|  |  | 15 | sA1/sA2 | sB1/sB1 | sC1/sC3 |
|  |  | 16 | sA1/sA1 | sB1/sB1 | sC2/sC3 |
|  |  | 17 | sA1/sA2 | sB1/sB1 | sC1/sC3 |
|  |  | 18 | sA2/sA2 | sB1/sB1 | sC1/sC1 |
|  |  | 19 | sA1/sA2 | sB1/sB1 | sC1/sC3 |
|  |  | 20 | sA1/sA2 | sB1/sB1 | sC1/sC3 |
|  |  | 21 | sA1/sA2 | sB1/sB1 | sC3/sC3 |
|  |  | 22 | sA1/sA2 | sB1/sB1 | sC1/sC1 |
|  |  | 23 | sA1/sA1 | sB1/sB1 | sC1/sC1 |
|  |  | 24 | sA1/sA1 | sB1/sB1 | sC2/sC3 |
| Putative hybrid | Wenchang | 1 | A_hyb1/A-hyb3 | B_hyb1/B_hyb3 | C_hyb1/C_hyb2 |
|  |  | 2 | A_hyb1/A-hyb3 | B_hyb1/B_hyb4 | C_hyb1/C_hyb2 |
|  |  | 3 | A_hyb1/A-hyb2 | B_hyb1/B_hyb4 | C_hyb1/C_hyb2 |
|  |  | 4 | A_hyb1/A-hyb2 | B_hyb1/B_hyb3 | C_hyb1/C_hyb2 |
|  |  | 5 | A_hyb1/A-hyb2 | B_hyb1/B_hyb3 | C_hyb1/C_hyb2 |
|  |  | 6 | A_hyb1/A-hyb2 | B_hyb1/B_hyb3 | C_hyb1/C_hyb2 |
|  |  | 7 | A_hyb1/A-hyb2 | B_hyb1/B_hyb3 | C_hyb1/C_hyb3 |
|  |  | 8 | A_hyb1/A-hyb2 | B_hyb2/B_hyb4 | C_hyb1/C_hyb2 |
|  |  | 9 | A_hyb1/A-hyb2 | B_hyb1/B_hyb3 | C_hyb1/C_hyb2 |
|  |  | 10 | A_hyb1/A-hyb3 | B_hyb2/B_hyb3 | C_hyb1/C_hyb2 |
|  | Suixi | 1 | A_hyb1/A-hyb2 | B_hyb1/B_hyb3 | C_hyb1/C_hyb2 |
|  |  | 2 | A_hyb1/A-hyb2 | B_hyb3/B_hyb5 | C_hyb1/C_hyb3 |
|  |  | 3 | A_hyb1/A-hyb2 | B_hyb1/B_hyb3 | C_hyb1/C_hyb2 |
|  |  | 4 | A_hyb1/A-hyb3 | B_hyb1/B_hyb3 | C_hyb1/C_hyb3 |
|  |  | 5 | A_hyb1/A-hyb3 | B_hyb1/B_hyb3 | C_hyb1/C_hyb2 |
|  |  | 6 | A_hyb1/A-hyb2 | B_hyb1/B_hyb3 | C_hyb1/C_hyb3 |
|  |  | 7 | A_hyb1/A-hyb2 | B_hyb1/B_hyb3 | C_hyb1/C_hyb2 |
|  |  | 8 | A_hyb1/A-hyb2 | B_hyb1/B_hyb3 | C_hyb1/C_hyb2 |
|  |  | 9 | A_hyb1/A-hyb2 | B_hyb3/B_hyb5 | C_hyb1/C_hyb3 |
|  |  | 10 | A_hyb1/A-hyb2 | B_hyb1/B_hyb3 | C_hyb1/C_hyb2 |
|  |  | 11 | A_hyb1/A-hyb2 | B_hyb3/B_hyb5 | C_hyb3/C_hyb4 |
|  |  | 12 | A_hyb1/A-hyb3 | B_hyb3/B_hyb5 | C_hyb1/C_hyb3 |

**Supplementary Table 2** Haplotypes at the three nuclear genes and their GenBank accession numbers for the three taxa in *Acrostichum*

| **Species** | **Gene** | **Haplotype** | **GenBank Accession No.** |
| --- | --- | --- | --- |
| *A. aureum* | *cam* | aA1 | JX575144 |
|  |  | aA2 | JX575145 |
|  | *gapcp1* | aB1 | JX575146 |
|  |  | aB2 | JX575147 |
|  |  | aB3 | JX575148 |
|  |  | aB4 | JX575149 |
|  |  | aB5 | JX575150 |
|  | *gapcp2* | aC1 | JX575151 |
|  |  | aC2 | JX575152 |
|  |  | aC3 | JX575153 |
|  |  | aC4 | JX575154 |
|  |  | aC5 | JX575155 |
|  |  | aC6 | JX575156 |
| *A. speciosum* | *cam* | sA1 | JX575165 |
|  |  | sA2 | JX575166 |
|  | *gapcp1* | sB1 | JX575167 |
|  |  | sB2 | JX575168 |
|  | *gapcp2* | sC1 | JX575169 |
|  |  | sC2 | JX575170 |
|  |  | sC3 | JX575171 |
| Putative hybrid | *cam* | A_Hyb1 | JX575141 |
|  |  | A_Hyb2 | JX575142 |
|  |  | A_Hyb3 | JX575143 |
|  | *gapcp1* | B_hyb1 | JX575157 |
|  |  | B_hyb2 | JX575158 |
|  |  | B_hyb3 | JX575159 |
|  |  | B_hyb4 | JX575160 |
|  |  | B_hyb5 | JX575161 |
|  | *gapcp2* | C_hyb1 | JX575162 |
|  |  | C_hyb2 | JX575163 |
|  |  | C_hyb3 | JX575164 |
